# The significance of peroxisome function in chronological aging of *Saccharomyces cerevisiae*

**DOI:** 10.1111/acel.12113

**Published:** 2013-07-08

**Authors:** Sophie D Lefevre, Carlo W van Roermund, Ronald J A Wanders, Marten Veenhuis, Ida J van der Klei

**Affiliations:** 1 Molecular Cell Biology, Groningen Biomolecular Sciences and Biotechnology Institute (GBB), University of GroningenP.O. Box 11103, 9700CC, Groningen, The Netherlands; 2 Departments of Pediatrics and Clinical Chemistry, Laboratory of Genetic Metabolic Diseases, Academic Medical Centre, University of Amsterdam1105 AZ, Amsterdam, The Netherlands

**Keywords:** chronological aging, energy metabolism, peroxisome, β-oxidation, yeast

## Abstract

We studied the chronological lifespan of glucose-grown *Saccharomyces cerevisiae* in relation to the function of intact peroxisomes. We analyzed four different peroxisome-deficient (*pex*) phenotypes. These included Δ*pex3* cells that lack peroxisomal membranes and in which all peroxisomal proteins are mislocalized together with Δ*pex6* in which all matrix proteins are mislocalized to the cytosol, whereas membrane proteins are still correctly sorted to peroxisomal ghosts. In addition, we analyzed two mutants in which the peroxisomal location of the β-oxidation machinery is in part disturbed. We analyzed Δ*pex7* cells that contain virtually normal peroxisomes, except that all matrix proteins that contain a peroxisomal targeting signal type 2 (PTS2, also including thiolase), are mislocalized to the cytosol. In Δ*pex5* cells, peroxisomes only contain matrix proteins with a PTS2 in conjunction with all proteins containing a peroxisomal targeting signal type 1 (PTS1, including all β-oxidation enzymes except thiolase) are mislocalized to the cytosol. We show that intact peroxisomes are an important factor in yeast chronological aging because all *pex* mutants showed a reduced chronological lifespan. The strongest reduction was observed in Δ*pex5* cells. Our data indicate that this is related to the complete inactivation of the peroxisomal β-oxidation pathway in these cells due to the mislocalization of thiolase. Our studies suggest that during chronological aging, peroxisomal β-oxidation contributes to energy generation by the oxidation of fatty acids that are released by degradation of storage materials and recycled cellular components during carbon starvation conditions.

## Introduction

Yeast are attractive eukaryote model organisms in aging studies, both for replicative lifespan and chronological lifespan (CLS) analysis. This is exemplified by the fact that several conserved factors involved in aging were first identified in yeast. In particular, the role of mitochondria has been extensively studied (Seo *et al*., 2010; Breitenbach *et al*., 2012). However, in aging studies, relatively little attention has been paid to the function of another important class of oxidative cell organelles, namely peroxisomes. Like mitochondria, peroxisomes produce reactive oxygen species (ROS), compounds which have been implicated to play an important role in aging.

Peroxisomes are single membrane-bound organelles that show an unprecedented variety of functions. Conserved functions are hydrogen peroxide metabolism and β-oxidation of fatty acids (Veenhuis *et al*., 1987; McCammon *et al*., 1990). Peroxisomes are dynamic organelles whose number and function are continuously adapted to cellular needs. Evidence has been presented that induction of peroxisome proliferation positively affects the lifespan of mice (Coschigano *et al*., 2003; Zhang *et al*., 2012). Also catalase, a conserved peroxisomal antioxidant enzyme, has been shown to contribute to lifespan determination in several model systems. The initial data on the role of peroxisomal catalase suggested that the absence of catalase decreases the lifespan of cells (Petriv & Rachubinski, 2004; Koepke *et al*., 2007, 2008). Recent studies however indicate that the absence of catalase also can extend the lifespan of cells at conditions that peroxisomal ROS activate anti-aging enzymes (Mesquita *et al*., 2010; Titorenko & Terlecky, 2011; Kawałek *et al*., 2013).

Here, we address the role of peroxisomes in chronological aging using the yeast *Saccharomyces cerevisiae*. In *S. cerevisiae,* peroxisome proliferation is induced during growth of cells on oleic acid. At these conditions, peroxisomes harbor enzymes of the β-oxidation cycle, acyl-CoA oxidase (Pox1), bifunctional enzyme (Fox2) and thiolase (Pot1), the key enzymes of the glyoxylate cycle citrate synthase (Cit2), malate dehydrogenase (Mdh3) and malate synthase (Mls1) as well as catalase A (Cta1) (van Roermund *et al*., 2003). Peroxisome biogenesis is controlled by a unique set of proteins termed peroxins, encoded by *PEX* genes (Nuttall *et al*., 2011). Deletion of *PEX* genes-encoding proteins involved in matrix protein import or formation of the peroxisomal membrane biogenesis results in the mislocalization of matrix proteins. As a consequence, *S. cerevisiae pex* mutants are unable to grow on oleic acid. However, these mutants normally grow on glucose, a carbon source that is not metabolized by peroxisomal enzymes.

So far, only few reports appeared on the role of peroxisomes in yeast aging. The chronological lifespan of two peroxisome-deficient mutant strains [Δ*pex5* (Goldberg *et al*., 2010) and Δ*pex6* (Jungwirth *et al*., 2008)] has been analyzed albeit at different cultivation conditions.

Jungwirth *et al*. (2008) showed that deletion of *PEX6* resulted in a decrease in survival of stationary phase cultures grown on synthetic complete (SC) media containing 2% glucose (Jungwirth *et al*., 2008). In cells lacking Pex6, all peroxisomal matrix proteins are mislocalized to the cytosol, but peroxisomal membrane proteins (PMPs) are correctly sorted to peroxisomal membrane remnants. When yeast cells are grown on 2% glucose, the acetic acid produced by the cells is a major cause of cell death. Acetic acid induces a mitochondrion-dependent apoptosis pathway (Ludovico *et al*., 2001; Burtner *et al*., 2009; Guaragnella *et al*., 2011). Why *PEX6* deletion resulted in a decreased lifespan and enhanced acetic acid sensitivity remained speculative (Jungwirth *et al*., 2008).

Goldberg *et al*. (2010) reported that deletion of *PEX5* reduces the CLS especially when low glucose concentrations (0.2 or 0.5%) were used (Goldberg *et al*., 2010). These authors used complex YP medium (1% yeast extract, 2% peptone) instead of SC medium.

Most peroxisomal matrix proteins contain a peroxisomal targeting signal 1 (PTS1), a tripeptide at the protein extreme C-terminus. Only few peroxisomal matrix proteins possess a peroxisomal targeting sequence 2 (PTS2). This signal is located at the N-terminus and has the consensus sequence (R/K)(L/V/I)X5(Q/H)(L/A/I). Pex5 and Pex7 are the receptor proteins for the PTS1 and PTS2, respectively. Deletion of *PEX5* leads to mislocalization of PTS1 proteins to the cytosol, whereas PTS2 proteins are still properly sorted to peroxisomes. Conversely, in Δ*pex7* mutants, PTS2 proteins remain in the cytosol, whereas PTS1 proteins are correctly imported into peroxisomes.

Peroxisomal acyl-CoA oxidase (Pox1), Fox2, Cit2, Mdh3, Mls1, and Cta1 contain a PTS1, whereas Pot1 has a PTS2. Hence, deletion of *PEX5* results in the mislocalization of most, but not all, enzymes of the β-oxidation pathway to the cytosol. Goldberg and colleagues suggested that the physical separation of the PTS2 protein Pot1 from the other β-oxidation enzymes in Δ*pex5* cells caused a block in this metabolic pathway, which could contribute to the reduced CLS (Goldberg *et al*., 2010).

*PEX5* and *PEX6* deletions give rise to different peroxisomal phenotypes (Nuttall *et al*., 2011), but it is unknown whether they have the same effect on yeast CLS. This led us to address the CLS of these deletion strains as well as of Δ*pex3* and Δ*pex7*, grown at the same conditions, namely mineral media containing 0.5% glucose. Δ*pex3* cells completely lack peroxisomal membranes, and hence, in these cells, all matrix proteins as well as PMPs are mislocalized. Our data indicate that β-oxidation is important for survival during chronological aging of *S. cerevisiae*.

## Results

### Peroxisome deficiency reduces the CLS of glucose-grown S. cerevisiae cells

**Fig. 1:**
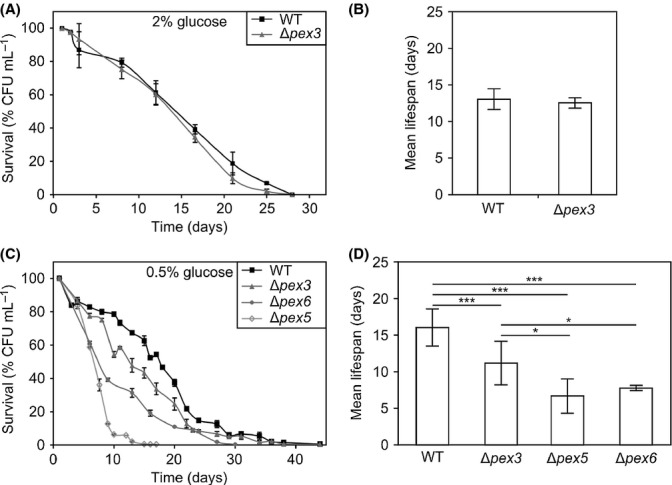
Peroxisome-deficient cells have a shorter lifespan than wild-type cells. (A) Chronological lifespan of WT and Δ*pex3* cells grown on 2% glucose. (B) The mean chronological lifespans of WT and Δ*pex3* cells calculated from the data in panel A. (C) Chronological lifespan of WT, Δ*pex3*, Δ*pex5,* and Δ*pex6* cells grown on 0.5% glucose. (D) The mean chronological lifespans of WT, Δ*pex3*, Δ*pex5,* and Δ*pex6* cells calculated from the data in panel C. Data represent mean ± SEM from at least three independent experiments. *P < 0.05; ***P < 0.005.

We first analyzed the CLS of a Δ*pex3* strain as this mutant shows the most severe peroxisome biogenesis defect. We did not observe significant differences in CLS (Fig. 1A,B, Table 1), upon growth of Δ*pex3* and wild-type (WT) control cultures on mineral media containing 2% glucose. At these conditions, the CLS of *S. cerevisiae* is mainly determined by acetic acid toxicity (Burtner *et al*., 2009). However, when Δ*pex3* cells were grown on mineral media containing 0.5% glucose, both the mean and maximal lifespan of the Δ*pex3* cultures were reduced (10.5 ± 2.98 days/22.9 ± 2.43 days) relative to the WT control (16 ± 2.52 days/26.6 ± 3.33 days) (Fig. 1C,D, Table 1). We therefore performed all subsequent CLS experiments with media containing 0.5% glucose.

**Table 1:** Mean and maximal lifespans

Strain	Mean CLS (days)	Maximal CLS (days)
2% glucose		
WT	13 ± 1.41	23.5 ± 0.71
*Δpex3*	12.5 ± 0.71	20.5 ± 0.71
0.5% glucose		
WT	16 ± 2.52	26.6 ± 3.33
Δ*pex3*	11.1 ± 2.98	22.9 ± 2.43
Δ*pex5*	6.7 ± 2.33	9.93 ± 2.07
Δ*pex6*	7.7 ± 0.35	21.5 ± 0.71
Δ*3'pex5*	7.5 ± 2.15	13.4 ± 1.63
Δ*pot1*	11.1 ± 1.44	20.4 ± 3.42
Δ*tgl3*	14.9 ± 1.27	24.5 ± 0.71
Δ*pex3*Δ*pot1*	8.6 ± 1.44	14.2 ± 2.17
Δ*pex7*	10.9 ± 0.14	20 ± 0.07
Δ*atg1*	15.5 ± 0.71	22.7 ± 0.35
Δ*atg1* Δ*pex3*	12 ± 0.01	19 ± 0.71
Oleic acid		
WT	36.5 ± 7.78	47 ± 2.83

The mean chronological lifespan is defined as the time point where 50% of the cells are viable. The maximum lifespan is the time point where 10% of the cells are viable.

Next, we analyzed two other *pex* mutants with other peroxisome biogenesis defects. In Δ*pex6* cells, PTS1 and PTS2 proteins are mislocalized to the cytosol, but peroxisomal membrane proteins (PMPs) are still correctly sorted to remnant peroxisomal membrane structures. In Δ*pex5* cells, only PTS1 proteins are mislocalized to the cytosol in conjunction with PMPs and PTS2 proteins localized to peroxisomes. As a consequence, the PTS1 and PTS2 enzymes of the β-oxidation pathway become physically separated in Δ*pex5* cells. As shown in Fig. 1C, the CLS of these two mutants is also shortened relative to the WT control upon growth on 0.5% glucose (Fig. 1C). Remarkably, Δ*pex5* cells displaying the mildest peroxisome biogenesis defect show the shortest CLS (6.7 ± 2.33 days/9.9 ± 2.07 days) (Fig. 1D, Table 1). Moreover, Δ*pex3* cells showing the strongest peroxisome biogenesis defect had a longer mean CLS relative to Δ*pex6* cells (7.8 ± 0.35 days vs. 10.5 ± 2.98 days), while the maximal lifespan is similar (21.5 ± 0.71 days vs. 22.9 ± 2.43 days) (Table 1).

**Fig. 2:**
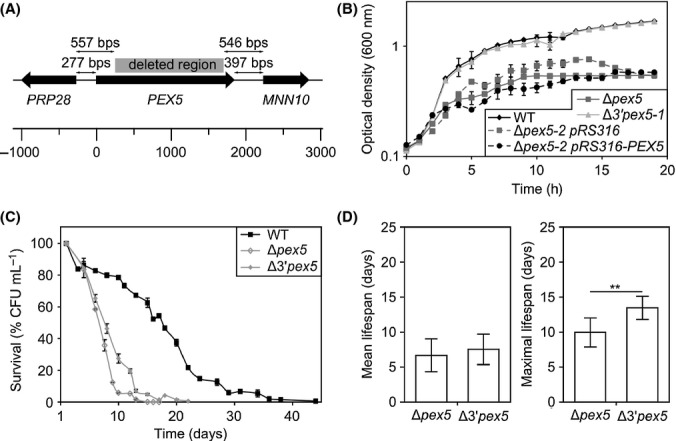
Deletion of *PEX5* by the Euroscarf strategy affects neighboring genes. (A) Genomic region of the *PEX5* gene. The distance between *PEX5* and *PRP28* or *MNN10* is 277 and 397 bps, respectively. Via the Euroscarf strategy, each gene was deleted from start to stop codon irrespectively of the genomic environment. A new strategy was designed to delete *PEX5* from +250 to +1700 leaving 557 and 546 bps for the *PRP28* and *MNN10* promoters, respectively. (B) Growth curves of different yeast strains on media containing 0.5% glucose. The optical density was determined as absorbance at 600 nm. Data represent mean ± SEM, from at least two experiments. (C) Chronological lifespan of Δ*pex5* and Δ*3'pex5* cells using WT as control. Data represent mean ± SEM from at least three experiments. (D) The mean and maximal chronological lifespans of Δ*pex5* and Δ*3'pex5* cells calculated from the data shown in panel C. **P < 0.01.

Unlike cultures of Δ*pex3* and Δ*pex6* cells, Δ*pex5* cultures showed a partial growth defect on glucose. Analysis of the genomic region of *PEX5* revealed that *PRP28* and *MNN10* are flanking the *PEX5* gene (Fig. 2A). *PRP28* encodes an essential RNA helicase involved in RNA isomerization at the 5′ splice site (Strauss & Guthrie, 1991; Staley & Guthrie, 1999). *MNN10* encodes a subunit of a Golgi mannosyltransferase complex the deletion of which affects growth (Karpova *et al*., 1993; Mondesert & Reed, 1996). In the used Δ*pex5* strain of the Euroscarf collection, the *PEX5* gene is deleted from start to stop codon. The short distance between *PRP28/MNN10* and *PEX5* does not exclude that this deletion strategy may have shortened the promoter region of one or both of the flanking genes and affect their transcription. To test this, we complemented the Euroscarf *PEX5* deletion strain by reintroducing the deleted *PEX5* gene on a plasmid (Δ*pex5/PEX5* strain). As shown in Fig. 2B, reintroduction of the *PEX5* gene did not result in restoration of the glucose growth defect. We also constructed a new *PEX5* deletion strain preserving at least 500 bps in front of the start codon of *PRP28* and *MNN10* to avoid truncation of the promoter regions of these two genes (Fig. 2A). This strain is named Δ*3'pex5*.

Growth experiments using different Δ*3'pex5* clones showed that the defect observed in growth of Δ*pex5* cells on glucose is abolished in all Δ*3'pex5* clones tested (Fig. 2B). This suggests that the transcription of either *PRP28* or *MNN10* or both was altered in the Euroscarf strain leading to an earlier entry into the stationary phase. Subsequently, we tested both Δ*pex5* and Δ*3'pex5* mutants for chronological aging. The maximal lifespan of the Δ*3'pex5* mutant is extended compared to the Euroscarf mutant (13.4 ± 1.63 days vs. 9.9 ± 2.07 days) indicating that the CLS of the Euroscarf strain is a result of a combination of the *PEX5* deletion in conjunction with other deficiencies (Fig. 2C, D, Table 1). Nevertheless, the lifespan of Δ*3'pex5* cells is still clearly reduced relative to WT cells and the shortest among all *pex* mutants under study, indicating that the defect in PTS1 protein import has a strong negative effect on chronological aging.

### Changes in autophagy do not explain the reduced lifespan of *pex* mutants

Increased autophagy during chronological aging enhances yeast chronological lifespan (Alvers *et al*., 2009; Eisenberg *et al*., 2009; Morselli *et al*., 2009, 2010; Aris *et al*., 2013). One explanation of the reduced lifespan in *pex* mutants may be that these mutations affect autophagy. To test this, we performed chronological aging experiments using Δ*atg1* and Δ*atg1*Δ*pex3* cells, strains in which autophagy is blocked. Upon deletion of *PEX3* in Δ*atg1* cells, both the mean and maximum lifespan decreases, indicating that also in the absence of autophagy, peroxisome deficiency reduces yeast lifespan (Fig. S1).

### Peroxisomes proliferate and the β-oxidation pathway is induced during chronological aging

**Fig. 3:**
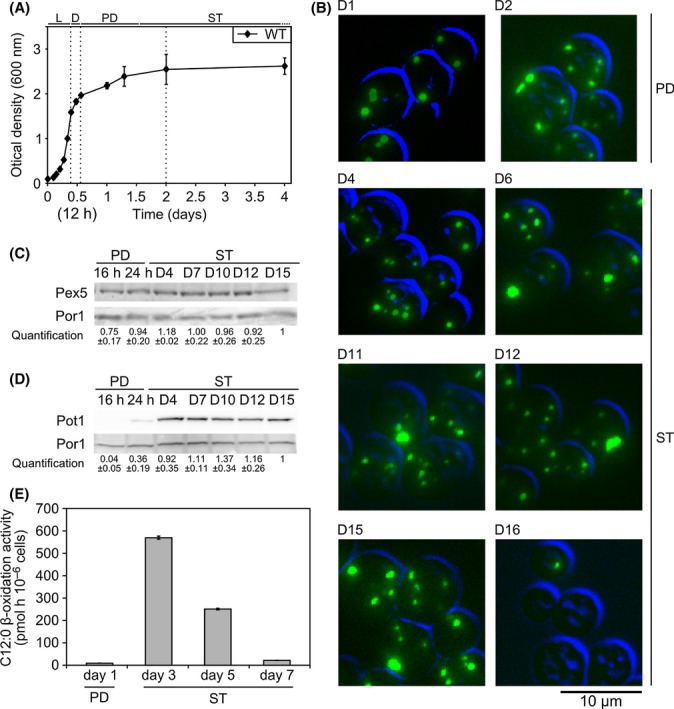
Peroxisome proliferation and β-oxidation during chronological aging of WT cells. (A) Growth curve of WT cells on 0.5% glucose. The data represent mean ± SEM from at least six independent experiments. L, logarithmic phase; D, diauxic phase; PD, postdiauxic phase; ST, stationary phase. (B) Fluorescence microscopy images of WT cells producing GFP-SKL to mark peroxisomes at different time points of a chronological aging experiment. The cell contours are indicated in blue. D-day. (C, D) Western blots, using total crude extracts, decorated with anti-Pex5 (C) or anti-Pot1 (D) antibodies showing the levels of these proteins during chronological aging. Antibodies against mitochondrial porine (Por1) were used as a loading control. The quantification reflects the relative amount of protein. The value at day 15 (D15) was set to 1. Differences in loading were corrected using quantification of the Por1 bands. Values were calculated from two different gels. (E) β-oxidation activities during chronological aging measured as the release of ^14^C-CO_2_ by a suspension of whole cells in the presence of ^14^C-lauric acid. The data represent mean ± SEM, *n* = 3.

In order to elucidate the principles of the shorter CSL of *pex* mutants relative to the WT control, as well as the mutual differences between the *pex* mutants studied, we investigated the fate of peroxisomes during chronological aging in WT cells. The growth curve of *S. cerevisiae* on 0.5% glucose starts with a short lag phase followed by logarithmic growth (2–10 h/0.4 days) and progresses through the diauxic shift (10–13 h/0.4–0.55 days) to the postdiauxic (13–48 h/0.55–2 days) and stationary phase (Fig. 3A). Using GFP-SKL as peroxisomal matrix marker, we observed that peroxisomes proliferate from 2.08 ± 0.87 per cell to 4.07 ± 1.29 per cell between 24 h (D1) and 48 h (D2), that is, during the late postdiauxic phase (Fig. 3B). Interestingly, in stationary phase cells, peroxisomes appear clustered in some cells, but remain detectable by fluorescence until 16 days (Fig. 3B).

We subsequently analyzed the levels of two proteins during chronological aging: Pex5 and Pot1, which are important for peroxisome biogenesis and function, respectively. Western blot analyses revealed that Pex5 is detectable at similar levels from day 1 (24 h) until day 15 (Fig. 3C). In contrast, Pot1 was very low during the growth phase (16 h after the start of the cultures the Pot1 level was 4% of that detected at day 15). This level rapidly increased during the postdiauxic phase (24 h after inoculation the pot level was 36% of the level detected at day 15 h). The highest level was detected at day 10, after which the levels slightly decreased (Fig. 3D).

In order to further analyze whether β-oxidation is induced during chronological aging of glucose-grown cells, we determined the activity of this pathway by the detection of CO_2_ production from radiolabelled lauric acid. These assays revealed that indeed, the β-oxidation pathway is induced in the early stationary phase (from day 3) (Fig. 3E), but drops to a low, basal level at day 7, although Pot1 protein levels were still relatively high at this stage.

### Peroxisomal β-oxidation supports the lifespan

The β-oxidation pathway converts activated fatty acids (fatty acyl-CoA) into NADH and acetyl-CoA that will subsequently be transferred to mitochondria to produce ATP. As sufficient ATP is essential to maintain cell viability, we tested whether peroxisomal β-oxidation contributes to survival of chronologically aging cultures and is responsible for the reduced CLS of glucose-grown cultures of *pex* strains.

**Fig. 4:**
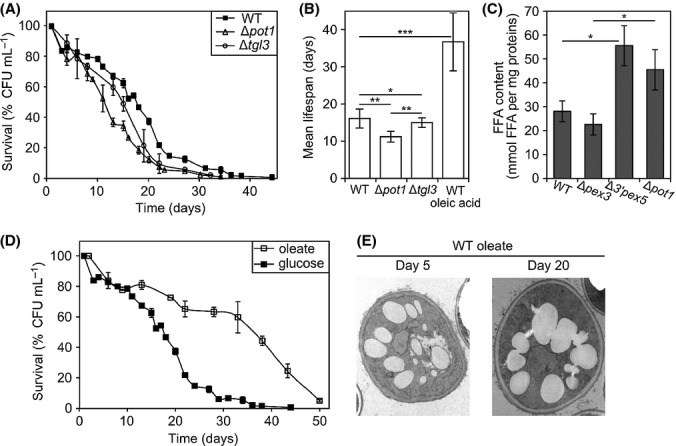
The significance of β-oxidation in chronological aging. (A) Chronological lifespan of Δ*pot1* and Δ*tgl3* cells relative to WT control cells. Cells were grown on 0.5% glucose. The data represent mean ± SEM from at least two experiments. (B) The mean chronological lifespans calculated from the data presented in panels A and D. *P < 0.05; **P < 0.01; ***P < 0.005. (C) Accumulation of free fatty acids (FFA) in WT, Δ*pex3*, Δ*3'pex5,* and Δ*pot1* cells at day 3 of a chronological aging experiment. *P < 0.05. (D) Chronological lifespans of WT cells grown on 0.5% glucose or 0.1% oleic acid. Data represent mean ± SEM from at least three experiments. (E) Transmission electron microscopy images showing the high content of lipid bodies in WT cells grown on oleic acid during chronological aging.

**Fig. 5:**
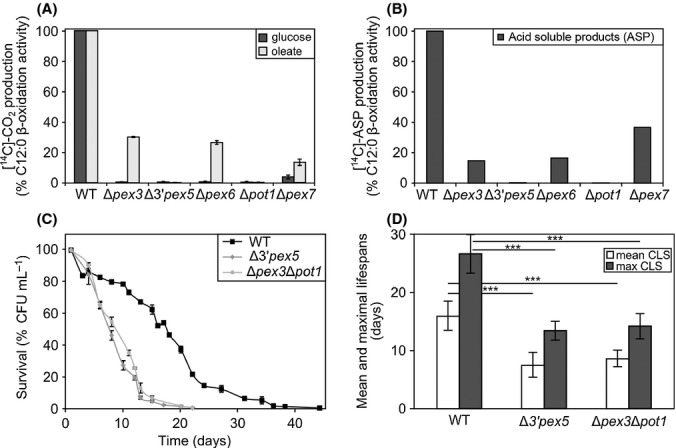
β-oxidation occurs in Δ*pex3* cells and is an important factor in chronological aging. (A) β-oxidation activity was measured using whole cells by determining the release of [^14^C]-CO_2_ during incubation in the presence of ^14^C-lauric acid. Cells were grown until OD = 1 on 0.5% glucose or in medium containing 0.1% oleic acid. Data are expressed as percentage of the WT activities, arbitrarily set to 100%. (B) β-oxidation activities measured by determining the levels of released ^14^C-labeled acid soluble products (ASP) in the presence of ^14^C-lauric acid. Cells were grown as indicated at A. Data are expressed as percentage of the WT activities, arbitrarily set to 100%. (C) Chronological lifespans of WT, Δ*pex3*Δ*pot1,* and Δ*3'pex5*. Data represent mean ± SEM from at least three experiments. (D) The mean and maximal chronological lifespans calculated from the curves presented in panel C.

First, we deleted the third enzyme of the β-oxidation pathway (3-ketoacyl-CoA thiolase, Δ*pot1*), which completely prohibits β-oxidation (see below; Fig. 5A,B). The mean and the maximal lifespan of Δ*pot1* cells are shortened (11.1 ± 1.44 days/20.4 ± 3.42 days) relative to WT controls (16 ± 2.52 days/26.6 ± 3.33 days; Fig. 4A,B, Table 1), indicating that β-oxidation is important for chronological aging.

During glucose growth, fatty acids are stored in lipid bodies as triacylglycerol (TG) and steryl esters (SE) (Henry *et al*., 2012). TG lipases and SE hydrolases are needed to mobilize them from these lipid bodies. Tgl3 is a TG lipase present at lipid bodies that converts TG to diacylglycerol (DG) plus free fatty acids (FFA) and also DG to monoacylglycerol (MG) plus FFA (Kurat *et al*., 2006). FFA can serve as substrates for peroxisomal β-oxidation (Henry *et al*., 2012). Deletion of *TGL3* slows down the mobilization of neutral lipids and hence the levels of FFA released from lipid bodies. As shown in Fig. 4A, the mean and maximum CLS of Δ*tgl3* cells are reduced (14.9 ± 1.27 days/24.5 ± 0.71 days) relative to WT cells (16 ± 2.52 days/26.6 ± 3.33 days), but the CLS decrease is less severe than in Δ*pot1* cells (11.1 ± 1.44 days/20.4 ± 3.42 days; Fig. 4A,B, Table 1).

The accumulation of FFA induces necrotic cell death (Rockenfeller *et al*., 2010). To test whether enhanced FFA levels in *pex* mutants explain their reduced lifespans, we determined FFA levels in the different mutant strains. FFA levels were measured at day 3 of the aging experiment, which corresponds to a time point of high β-oxidation activity in WT cells (Fig. 3E). As shown in Fig. 4C, FFA levels were similar in Δ*pex3* and WT cells. Hence, the reduced lifespan of Δ*pex3* cells cannot be explained by enhanced FFA levels. Moreover, the FFA levels in Δ*3'pex5* and Δ*pot1* are similar, but the lifespan of Δ*pex5* cells is much shorter relative to that that of Δ*pot1* cells. These data suggest that lipid toxicity had no major effect on the lifespan of the cultures under the experimental conditions used.

To test whether indeed β-oxidation enhances yeast CLS, we performed aging experiments using WT cells grown on oleic acid, a substrate that induces enzymes of the β-oxidation pathway. As shown in Fig. 4B,D and Fig. S2, culturing WT cells on oleic acid significantly extends both the mean and maximum lifespan (see also Table 1). In these cells, massive amounts of lipid bodies accumulate (Fig. 4E), which can supply substrates for peroxisomal β-oxidation during chronological aging.

However, the CLS of mutants showing strong defects in β-oxidation is extremely short upon growth in the presence of oleic acid (Fig. S2). This is associated with a strong increase in FFA levels (Fig. S2B), which could lead to lipotoxicity.

### The peroxisomal β-oxidation pathway is functional in Δ*pex3* and Δ*pex6* cells but not in Δ*pex5* cells

The above data suggest that β-oxidation may be important to support the lifespan; alterations in the activity of this pathway may modulate CLS of peroxisome-deficient cells. In Δ*pex3* and Δ*pex6* cells, all enzymes of the β-oxidation pathway are cytosolic, whereas in Δ*pex5* cells, only Pox1 and Fox2 are cytosolic while the PTS2 protein Pot1 is normally imported in peroxisomes. This leads to a physical partitioning of these enzymes.

When cells were grown on glucose until the late exponential phase, β-oxidation activities were very low in all strains. In WT cells, β-oxidation was still detectable, but in the *pex* mutants, the activities of this pathway were undetectable or at the limit of detection (measured as production of [^14^C]-CO_2_ from ^14^C-lauric acid). Growth on oleic acid resulted in a strong increase in β-oxidation activity in WT cells. Growth in the presence of oleic acid also resulted in an increase in β-oxidation activities in Δ*pex3* and Δ*pex6* cells, but not in Δ*pex5* cells. Like in the Δ*pot1* control strain, the β-oxidation activity in Δ*pex5* cells was at the limit of detection (Fig. 5A).

To exclude that low β-oxidation activity, measured as production of [^14^C]-CO_2_ from ^14^C-lauric acid, was related to a defect in mitochondrial respiration, we also measured the production of ^14^C-labeled acid soluble products (ASP) from ^14^C-lauric acid. ASPs are direct products of the β-oxidation pathway. As shown in Fig. 5B, the production of ASPs followed the same trend as [^14^C]-CO_2_ production (Fig. 5B).

### Inactivation of β-oxidation in Δ*pex5* cells contributes to decrease cell viability

To test whether the differences in β-oxidation in Δ*pex3* and Δ*pex5* cells explain the differences in chronological aging, we blocked the residual β-oxidation activity in Δ*pex3* creating a Δ*pex3*Δ*pot1* double deletion strain. The CLS of Δ*pex3*Δ*pot1* was reduced compared to Δ*pex3* (Fig. 5C, Table 1). Moreover, the mean and maximal lifespans of Δ*pex3*Δ*pot1* are similar to that of Δ*3'pex5*. This suggests that indeed, the difference in CLS of Δ*pex3* and Δ*3'pex5* cells is related to inactivation of the β-oxidation pathway in Δ*pex5* cells (Fig. 5D, Table 1).

### Deletion of *PEX7* results in a decreased CLS

**Fig. 6:**
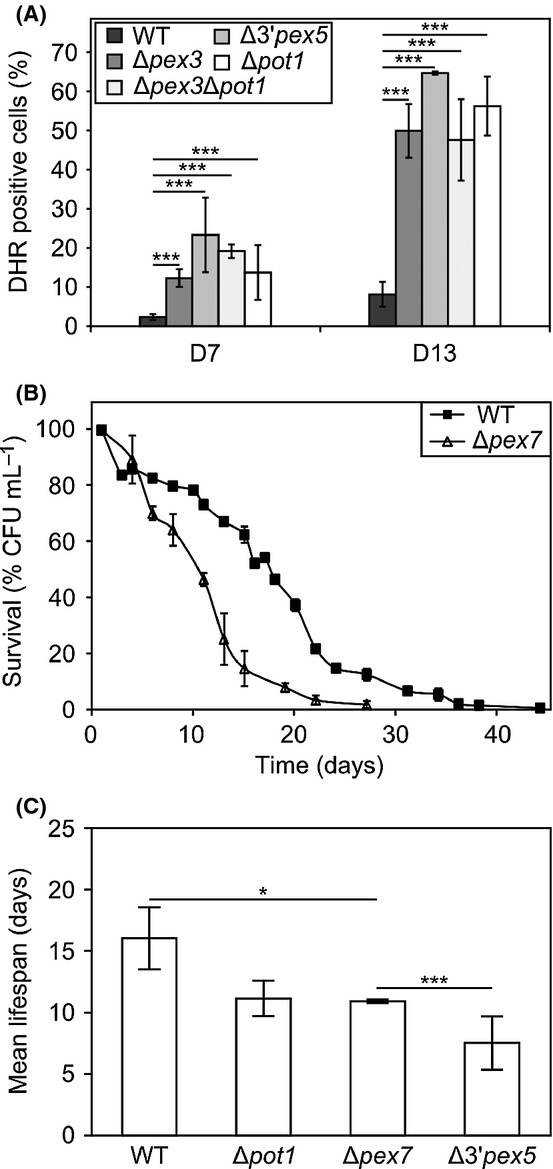
Deletion of *PEX7* shortens the lifespan. (A) FACS measurements of H_2_O_2_ accumulation using dihydrorhodamine 123 in WT, Δ*pex3*, Δ*3'pex5*, Δ*pex3*Δ*pot1,* and Δ*pot1* at day 7 (dark gray) and day 13 (light gray). The graph indicates percentages of positive cells ± SD measured in 20 000 cells per sample from two independent experiments. ***P < 0.005. (B) Effect of *PEX7* deletion on chronological lifespan. (C) Mean chronological lifespans of WT and mutant cells. Data represent mean ± SEM from at least two experiments. *P < 0.05; ***P < 0.005.

The lifespan of Δ*pot1* cells (Fig. 4A) is longer compared to that of Δ*pex3*Δ*pot1* or Δ3'*pex5* cells (Table 1). Because in all three strains, the β-oxidation is blocked, additional processes most likely contribute to the reduction of the lifespan of peroxisome-deficient strains. In all three mutant strains, the initial oxidation of fatty acyl-CoA (catalyzed by Pox1) can still occur. This reaction results in the production of hydrogen peroxide, which is decomposed by peroxisomal catalase (Cta1) in WT cells. In Δ*pex3*Δ*pot1*, Δ3'*pex5* and Δ*pex3*, where both Pox1 and Cta1 are mislocalized to the cytosol, cells accumulate comparable levels of H_2_O_2_ during chronological aging as in Δ*pot1* cells, in which both enzymes are localized to peroxisomes (Fig. 6A). Hence, the observed differences in CLS of Δ*pot1* cells and the various *pex* mutants are unlikely due to differences in cellular H_2_O_2_ levels.

To determine whether cytosolic mislocalization of only PTS1 proteins contributes to the relatively short CLS of *pex* strains, we also analyzed chronological aging of a Δ*pex7* culture in which thiolase is located in the cytosol. Deletion of *PEX7* indeed resulted in a reduction in β-oxidation activity, however not to a complete block (Fig. 5A,B). The lifespan of Δ*pex7* cells was similar to that of other *pex* mutants that show residual β-oxidation activity (Table 1).

## Discussion

We have analyzed the CLS of *Saccharomyces cerevisiae* WT cells relative to peroxisome-deficient (*pex*) mutants that have different peroxisome biogenesis defects ranging from the complete absence of peroxisomes (Δ*pex3*), a defect in import of all matrix proteins, but not of PMPs (Δ*pex6*), to a defect in import of either PTS1 (Δ*pex5*) or PTS2 proteins (Δ*pex7*).

First, we compared the CLS of WT and mutant cells with the most severe peroxisome biogenesis defect (Δ*pex3*) on media containing 2% glucose. Our data indicate that deletion of *PEX3* did not affect the CLS under these conditions (Fig. 1A,B). This is most likely related to the fact that when cells are grown on media containing high glucose concentrations, medium acidification is the major cause of CLS reduction in yeast (Burtner *et al*., 2009).

Subsequently, we lowered the glucose concentration to 0.5%, which is known to result in extension of yeast CLS (MacLean *et al*., 2001; Oliveira *et al*., 2008; Matecic *et al*., 2010). In this medium, we indeed observed a lifetime extension for WT cells (Fig. 1C,D). However, all *pex* mutants showed a reduced CLS compared to the WT control, indicating that the peroxisome biogenesis defects affect the CLS when cells are grown on 0.5% glucose.

Autophagy is an important factor in chronological aging. Reduction in autophagy results in a shortening of the CLS, whereas stimulation of autophagy extends the lifespan (Alvers *et al*., 2009; Eisenberg *et al*., 2009; Morselli *et al*., 2009, 2010; Aris *et al*., 2013). Because deletion of *PEX3* in Δ*atg1* cells, in which autophagy is blocked, also resulted in a lifespan reduction (Fig. S1), the observed negative effects of *pex* mutations are not indirectly due to changes in autophagy.

Our data revealed that Δ*pex5* cells showed the shortest lifespan among all tested *pex* mutants (Δ*pex3,* Δ*pex5,* Δ*pex6,* and Δ*pex7*) (Fig. 1C,D; Table 1). It has been suggested that a block in β-oxidation due to the segregation of thiolase (Pot1) from the other β-oxidation enzymes explains the reduced CLS of Δ*pex5* cells (Goldberg *et al*., 2010). Activity measurements showed that β-oxidation was indeed fully blocked in Δ*pex5* cells, but not in Δ*pex3* and Δ*pex6* cells (Fig. 5A,B).

Unexpectedly, we also detected residual β-oxidation activity in Δ*pex7* cells (Fig. 5 A,B). One explanation could be that some thiolase molecules are still imported into peroxisomes, possibly by piggybacking with a PTS1 matrix protein (Horiguchi *et al*., 2001; Islinger *et al*., 2009). Alternatively, the thiolase substrate, 3-ketoacyl-CoA, formed inside peroxisomes of Δ*pex7* cells, might be exported to the cytosol where it can be cleaved by thiolase. However, the existence of such peroxisomal transporters is not yet reported.

Our analysis of WT cells demonstrated that indeed β-oxidation and peroxisome proliferation are induced during the postdiauxic phase and stationary phase of the glucose cultures (Fig. 3B,E). During the stationary phase, cells consume storage compounds to produce energy when glucose and ethanol are exhausted. During degradation of lipid bodies and phospholipids derived from membranes upon autophagic degradation of organelles, free fatty acids will be released. These are the substrates for peroxisomal β-oxidation to produce energy. The β-oxidation pathway converts fatty acyl-CoA to acetyl-CoA and NADH. Acetyl-CoA is exported from peroxisomes via the carnitine shuttle or glyoxylate cycle and is ultimately further oxidized in the TCA cycle resulting in NADH generation that can be further used to produce ATP via the respiratory chain. Intracellular ATP levels are directly correlated with the viability during aging and are a decisive factor for the cell survival (Goldberg *et al*., 2009). Our observation that β-oxidation activity peaks at the beginning of the stationary phase supports the fact that peroxisomal β-oxidation actively participates to fulfill cellular energy requirements at this stage (Fig. 3E).

Consistent with this, a block in β-oxidation explains the reduction in the CLS of glucose-grown yeast cultures. β-oxidation is fully blocked in Δ*pex5* and Δ*pot1* cells (Fig. 5A,B). However, the CLS of Δ*pex7* and Δ*pot1* cultures is similar, but extended relative to that of Δ*pex5* cultures (Fig. 6 B,C). This suggests that the reduced levels of β-oxidation activity in Δ*pex3*, Δ*pex6,* and Δ*pex7* cells do not fulfill the energy requirements during aging.

Because a full block in β-oxidation (in Δ*pot1* cells) has a less severe effect on aging that deletion of *PEX5*, which also causes a full block in β-oxidation, other still unknown peroxisomal processes are also important for chronological aging. The fact that in Δ*pex3* and Δ*pex6,* PTS1 proteins are also mislocalized probably explains the reduced CLS of these strains, despite the fact that the cells display a residual β-oxidation activity. This β-oxidation activity in Δ*pex3* cells is indeed relevant for chronological aging, because a Δ*pex3*Δ*pot1* double mutant has a shorter CLS relative to a Δ*pot1* mutant but equal to that of Δ*pex5* cells (Fig. 5C; Table 1). As in the all three strains, β-oxidation is completely blocked, additional peroxisomal processes may play a role in determining the reduced CLS in *pex* cells.

Most likely, this is not due to oxidation of fatty acyl-CoA in the cytosol by Pox1, which results in the release of hydrogen peroxide in the cytosol. Although the peroxisomal catalase is mislocalized to the cytosol in Δ*pex3*, Δ*pex5,* and Δ*pex6* cells, cytosolic anti-oxidant enzymes can detoxify Pox1-driven hydrogen peroxide production as efficiently as Cta1 in peroxisomes in Δ*pot1* cells (Fig. 6A). Hence, ROS-related damages do not explain the different lifespans.

Alternatively, the glyoxylate cycle, the key enzymes of which are localized to peroxisomes (malate dehydrogenase) and the cytosol (isocitrate lyase), may function less efficient when fully localized to the cytosol. The glyoxylate cycle generates succinate from two molecules of acetyl-CoA, which is important for the generation of carbohydrates and precursors of amino acids during growth of *S. cerevisiae* on oleic acid or ethanol. It can be envisaged that the reduced efficiency of the glyoxylate cycle in the cytosol affects growth in the postdiauxic phase and as such account for the reduced lifespan of Δ*pex3*Δ*pot1* and Δ*pex5* cells, relative to the single *POT1* deletion CLS during the stationary phase.

Reduced β-oxidation activity may also lead to the accumulation of FFAs that are highly toxic and induce cell death (Jungwirth *et al*., 2008; Rockenfeller *et al*., 2010). When *pex* mutants are grown on oleic acid-containing medium, we indeed observed a rapid loss of viability associated with high FFA levels (Fig. S1). However, when cells are grown on low medium containing 0.5% glucose, FFA accumulation is low (Fig. 4C). Because the same FFA levels were observed in Δ*pex5* cells and in Δ*pot1* cells, it is unlikely that this affected the lifespan, because Δ*pot1* cells live longer that Δ*pex5* cells (Fig. 4C). Therefore, we conclude that lipotoxicity due to FFA accumulation does not explain the reduced lifespans of *pex* mutants in glucose-grown cultures.

Finally, we showed that the Δ*pex5* strain from the Euroscarf collection has an additional defect, due to interference with the promoter regions of flanking genes. Unlike Δ*pex5/PEX5* cells, our new Δ*pex5* strain did not show this defect (Fig. 2). Similar phenomena may occur in other strains of the Euroscarf collection and should be taken in consideration carefully.

## Experimental procedures

### Strains and growth conditions

The yeast strains used in this study are listed in Table S1. *Saccharomyces cerevisiae* was grown on mineral medium (MM) (van Dijken *et al*., 1976) containing 0.25% ammonium sulfate, 0.05% yeast extract and 2% or 0.5% glucose. For peroxisome induction, MM containing 0.02% yeast extract, 0.1% oleic acid, and 0.05% Tween 80 was used. MM was supplemented with the required amino acids to a final concentration of 20 μg mL^−1^ (histidine) or 30 μg mL^−1^ (leucine, lysine, and uracil). For growth on agar plates, the YPD medium (1% yeast extract, 1% peptone, 1% glucose) was supplemented with 2% agar. *Escherichia coli* DH5α was used for plasmid constructions and was cultured on LB medium supplemented with the appropriate antibiotics.

For *PEX5* complementation (Δ*pex5/PEX5* strain), the *PEX5* open reading frame plus 487-bps upstream the start codon and 443 bps after the stop codon was amplified using primers Pex5.5 and Pex5.6 (Table S2). After *Xba*I digestion, the 3056-bps PCR fragment was cloned into pRS316. Δ*pex5* cells (Euroscarf collection) were transformed with this centromeric plasmid, and positive clones were selected on YNB plates (BD Difco™, BD Biosciences, San Jose, CA, USA) supplemented with 2% glucose and 20 μg mL^−1^ histidine, 30 μg mL^−1^ leucine, and 30 μg mL^−1^ lysine. Successful transformation was checked by colony PCR using primers Pex5.1 + Pex5.2 and Pex5.A + Pex5.B (Table S2).

The Δ*3'pex5* deletion strain was constructed by replacing the +250 to +1700 genomic region of *PEX5* by the antibiotic marker kanamycin. A PCR-based deletion strategy was used, and primers were designed to have a tail of 50 nucleotides homologous to the *PEX5*-targeted region. The PCR fragment was obtained by amplification of the *loxP-KanMX-loxP* cassette from pUG6 (Güldener *et al*., 1996) using the primers Pex5UP and Pex5DN (Table S2). Subsequently, *S. cerevisiae* wild-type cells were transformed with the 1713-bps *pex5::loxP-KanMX-loxP* DNA fragment, and clones were selected on YPD agar plates containing 200 μg mL^−1^ G418. Successful integration by homologous recombination was checked using the primers Pex5.1, Pex5.2 (Table S2), and the Euroscarf (EUROSCARF, European *Saccharomyces cerevisiae* Archive for Functional Analysis, Institut für Mikrobiologie, Johann Wolfgang Goethe-Universität, Frankfurt, Germany) confirmation PCR primers KanB and KanC (Winzeler *et al*., 1999).

*POT1* was deleted in Δ*pex3* cells by replacing the open reading frame of the gene by the nourseothricin resistance gene, *NatMX4* (Goldstein & McCusker, 1999). To this purpose, two DNA fragments comprising the regions −536 to −26 and +226 to +2615 of the *POT1* genomic region were amplified using the primers Pot1.1 + Pot1.2 and Pot1.3 + Pot1.4 (Table S2) and *S. cerevisiae* gDNA as template. PCR fragments were digested with *BamH*I/*Pvu*II and *Sac*II/*Spe*I, respectively, and subcloned into pAG25 (Goldstein & McCusker, 1999) on both sides of the *NatMX4* selection marker to product pSL38 plasmid. This plasmid was digested using *Sac*II and *Pvu*II restriction enzymes and the DNA fragment *pot1::NatMX4* was used to transform Δ*pex3* cells. For the selection of positive clones, YPD plates containing 100 μg mL^−1^ nourseothricin (Invitrogen, Carlsbad, CA, USA) were used. Correct integration was checked using the primers Pot1.5, Pot1.6, Pot1.7, and Pot1.8 (Table S2).

Peroxisomes were labeled by expressing green fluorescent protein containing a peroxisomal targeting signal (GFP.SKL) under the control of the *MET25* promoter. The P_*MET25*_-GFP.SKL-T_*CYC1*_ fragment from plasmid pRS6MPTS1GFP (Schäfer *et al*., 2004) was amplified using the primers GFPSKL-3 and GFPSKL-4 (Table S2) and subsequently digested with *Sac*II and *Not*I. This DNA fragment was subcloned into pHIPZ plasmid (laboratory collection) carrying the zeocin resistance gene and no yeast origin of replication. The resulting integrative plasmid was named pSL34. This plasmid was then digested by *Nar*I, allowing genomic integration into the *MET25* promoter. Correct integration was checked using the MET25.1 and GFPSKL-3 primers (Table S2).

### Chronological lifespan and growth measurements

In a standard experiment, overnight cultures were grown in MM medium containing 0.5% glucose. The cultures were then diluted twice at OD_600 nm_ = 0.1 in fresh MM and grown for 8 h. After the last precultivation step, cells were diluted in MM containing either glucose or oleic acid. Cultures were incubated at 30 °C, 200 rpm. Optical density was monitored at 600 nm. Survival was assayed by counting colony-forming units (CFUs) after 2 days of incubation at 30 °C on YPD agar plates. Survival at 24 h after the last dilution (D1) was set to 100% of survival. Mean values are presented together with the standard error of mean. Statistical analyses were performed using two-way ANOVA. A *P*-value of < 0.05 was considered as a significant difference.

### Fluorescence microscopy

An Axio Observer Z1 fluorescence microscope (Carl Zeiss, Oberkochen, Germany) equipped with a 470/40-nm bandpass excitation filter and a 525/50-nm bandpass emission filter was used to visualize GFP. A Coolsnap HQ2 digital camera (Roper Scientific Inc., Trenton, NJ, USA) was used. Images were processed using metavue software (Molecular Devices Metavue, Dowington, PA, USA) and analyzed using image j software (US National Institutes of Health, Bethesda, MD, USA).

### TCA protein extraction and Western blotting

10^8^ cells were harvested and washed in water, and extracts were prepared by disrupting the cells in 1.85 N NaOH and 2% β-mercaptoethanol. Proteins were precipitated with 12.5% TCA. After centrifugation, pellets were resuspended in Laemmli sample buffer for 5 min at 100 °C. Proteins were separated on 12.5% SDS-polyacrylamide gels and transferred to a nitrocellulose filter. Western blotting was performed using Pex5, Pot1, and Por1 antibodies. Pex5/Por1 blots were decorated using alkaline phosphatase-conjugated anti-rabbit secondary antibody (Roche Applied Science, Almere, the Netherlands) and NBT/BCIP (nitro blue tetrazolium chloride/5-bromo-4-chloro-3-indolyl phosphate, Roche Applied Science). Pot1/Por1 blots were decorated using horseradish peroxidase-conjugated anti-rabbit secondary antibody (Santa Cruz Biotechnology Inc., Santa Cruz, CA, USA) followed by chemiluminescence detection (Amersham ECL Prime Western Blotting Detection Reagent, Amersham Biosciences Europe GmbH, Freiburg, Germany). Quantifications were performed using imagej software. The anti-Pex5 and anti-Pot1 polyclonal antibodies were a gift from prof. R. Erdmann, Bochum, Germany. Anti-Por1 was a gift from Dr. M. van der Laan, University of Freiburg.

### β-oxidation measurements

Glucose-grown WT cells were harvested at day 1, 3, 5, and 7 of the aging experiments. For measurements using cells of *pex* mutants, cells cultivated in the presence of oleate were collected from cultures in the late exponential growth phase. β-oxidation assays were performed using intact cells basically as described by van Roermund *et al*. (van Roermund *et al*., 2008) with slight modifications. β-oxidation (CO_2_ production) was measured in 50-mM MES, pH 6.0 supplemented with 10 μm ^14^C-lauric acid (C12:0). Subsequently, the produced [^14^C]-CO_2_ was trapped with 2 m NaOH and used to quantify the rate of fatty acid oxidation per 10^6^ cells. To detect the production of acyl-CoA esters, the reaction was stopped by incubating the cells with 2.6 m perchloric acid. Hydrolysis of CoA esters was performed by incubation in 2 m NaOH at 50 °C followed by incubation in a sodium acetate buffer pH 6.0 and 0.5 m H_2_SO_4_. Fatty acids were then extracted, and acid soluble products were measured with a phosphorimager (van Roermund *et al*., 1998). Results are expressed as percentages relative to the rate of β-oxidation in WT cells.

### FACS analysis of intracellular levels of hydrogen peroxide

Levels of intracellular H_2_O_2_ were measured using dihydrorhodamine 123 (DHR) (Molecular Probes, Eugene, OR, USA). Briefly, samples of the cultures were taken at selected time points, and DHR was added to a final concentration of 20 μm. Cells were incubated for 30 min at room temperature in the dark. Cells were subsequently washed twice in PBS and analyzed by flow cytometry. FACS analysis was performed using an FACS Aria II Cell sorter (BD Biosciences). Fluorescence signal of individual cells was captured in a FACS Aria II Cell sorter (BD Biosciences) for 20 000 events a 488-nm laser, 505-nm long pass mirror, and 525/50-nm band-pass filter. facsdiva software version 6.1.2 (BD Biosciences, Breda, the Netherlands) was used for data acquisition and analysis.

### Electron microscopy

Cells were collected by centrifugation and fixed in 1.5% (w/v) KMnO_4_ for 20 min at room temperature. Cells were then incubated overnight in 1% uranyl acetate (w/v), dehydrated in a graded ethanol series, and embedded in Epon 812. Ultrathin sections were studied in a Philips electron microscope CM-12.

### Analysis of the levels of free fatty acids

Cells were broken with sterile glass beads in 50 mm potassium phosphate buffer pH 7.4. Nonbroken cells were removed by centrifugation. Free fatty acids (nonesterified fatty acids) were detected using the Free Fatty acids Half-micro test kit (Roche Applied Science) according to the instructions of the manufacturer. Protein concentrations were determined using the Bradford assay. FFA accumulation was expressed in mmol FFA/mg protein.

## Supporting Information

**Fig. S1** Lifespan reduction in *pex* mutants is independent on macroautophagy.

**Fig. S2** Chronological aging of *pex* mutants on oleic acid-containing medium.

**Table S1** Yeast strains used in this paper.

**Table S2** Primers used in this paper.

**Data S1** Experimental procedures.
