# Supplementary figures and images for: The significance of peroxisome function in chronological aging of Saccharomyces cerevisiae
Source: Aging Cell. 2013 Jul 8;12(5):784–93. doi: 10.1111/acel.12113 (PMC3824234; doi:10.1111/acel.12113)

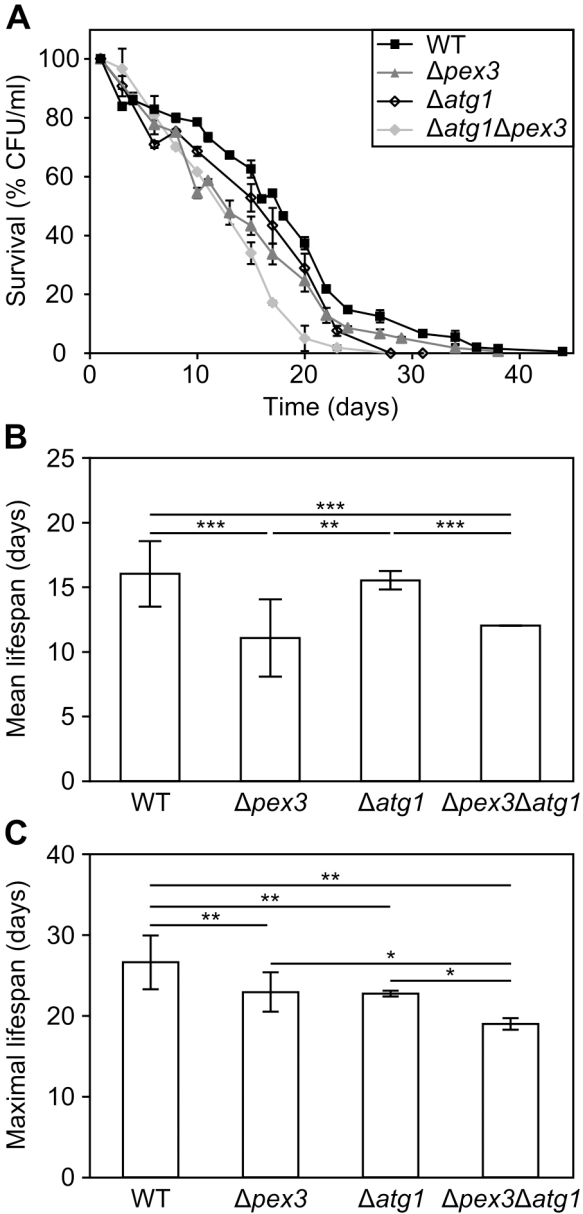

Figure S1

Supplement: Supplementary file 1 [file acel0012-0784-SD1.pdf]

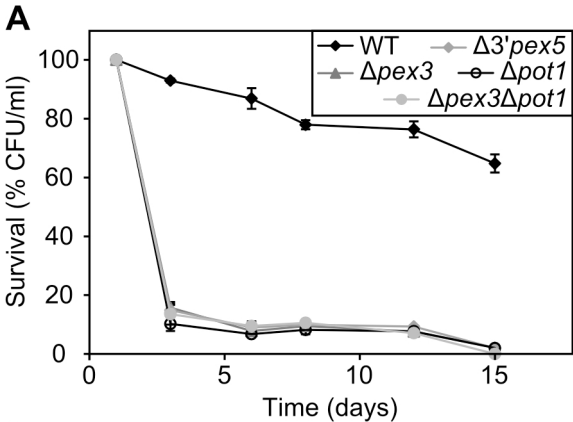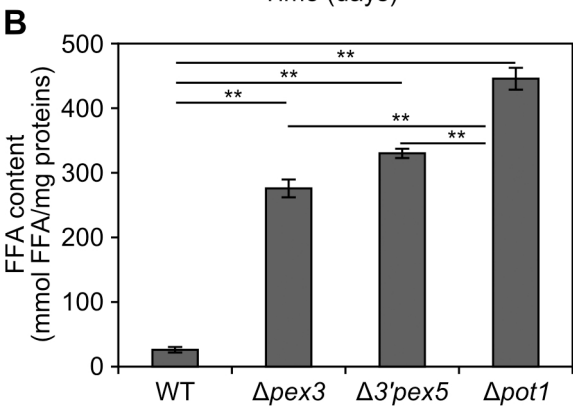

Figure S2

Supplement: Supplementary file 2 [file acel0012-0784-SD2.pdf]
