# Supplementary material for: The significance of peroxisome function in chronological aging of Saccharomyces cerevisiae
Source: Aging Cell. 2013 Jul 8;12(5):784–93. doi: 10.1111/acel.12113 (PMC3824234; doi:10.1111/acel.12113)
Supplement: Supplementary file 3 [file acel0012-0784-SD3.docx]

**Supporting information**

**Supplementary tables**

**Table S1:** Yeast strains used in this paper.

| **Strains** | **Description** | **Reference** |
| --- | --- | --- |
| WT | BY4742 MATα *his3*Δ*1 leu2*Δ*0 lys2*Δ*0 ura3*Δ*0* |  |
| Δ*pex3* | BY4742 Δ*pex3::KanMX4* | Euroscarf collection |
| Δ*pex5* | BY4742 Δ*pex5::KanMX4* | Euroscarf collection |
| Δ*pex6* | BY4742 Δ*pex6::KanMX4* | Euroscarf collection |
| Δ*3’pex5* | BY4742 Δ*3’pex5::KanMX4* | this study |
| Δ*pot1* | BY4742 Δ*pot1::KanMX4* | Euroscarf collection |
| Δ*tgl3* | BY4742 Δ*tgl3::KanMX4* | Euroscarf collection |
| Δ*pex3*Δ*pot1* | BY4742 Δ*pex3::KanMX4* Δ*pot1::NatMX4* | this study |
| Δ*pex7* | BY4742 Δ*pex7::KanMX4* | Euroscarf collection |
| WT GFP.SKL | BY4742 *pMET25-GFP.SKL/Zeo^R^* | this study |
| Δ*atg1* | BY4741 Δ*atg1::KanMX4* | Euroscarf collection |
| Δ*atg1*Δ*pex3* | BY4741 Δ*pex3::KanMX4* Δ*atg1::NatMX4* | this study |
| Δ*pex5/PEX5* | BY4742 Δ*pex5::KanMX4* pRS316-*PEX5* | this study |

**Table S2:** Primers used in this paper.

| **Primers** | **Sequence 5’-3’** |
| --- | --- |
| Pex5UP | TATGCAAAGGTTCATAAACGGAGAACCACTGATCGATGATAAAAGAAGAA-CAGCTGAAGCTTCGTACGC |
| Pex5DN | CTCTCTTCAAAGTCTCTATAACAGTATCATTGTACGTATTCAAGAGAGAT-GCATAGGCCACTAGTGGATCTG |
| Pex5.1 | GGCGTCTTAATGAGTCACCT |
| Pex5.2 | ATGCCTGGCTTCACTTCTTG |
| Pex5.5 | ATCCGCTCAGAGTATCTTCG |
| Pex5.6 | TCCATGTCTCTTCGCATAGG |
| Pex5.A | GCTTGCTGATTTTACCTGATGTATT |
| Pex5.B | GAGAGCTTTTCTCTCCCTGATAAAC |
| Pot1.1 | CTACAGCTGCTAACGCTACACCGACCAA |
| Pot1.2 | CTAGGATCCCTGTACTCAGAGCCACAAG |
| Pot1.3 | CTAACTAGTGCCGCCGCCATCTT |
| Pot1.4 | CTACCGCGGACGTTACCTCATATGGCTATCG |
| Pot1.5 | GAGGCATGCACTTCGGATTA |
| Pot1.6 | AATTCAACGCGTCTGTGAGG |
| Pot1.7 | GACATCATCTGCCCAGATGC |
| Pot1.8 | TGGAGGGGAAGAAGTGAGAG |
| GFPSKL-3 | TATCCGCGGCGCGCAATTAACCCTCA |
| GFPSKL-4 | TATGCGGCCGCGTAACGCCAGGGTTTT |
| MET25.1 | GGCGTCAGATTTAGGTGGAT |
| ATG1up | TTCAAATCTCTTTTACAACACCAGACGAGAAATTAAGAAA-GACGGATCCCCGGGTTAATTA |
| ATG1down | GGTCATTTGTACTTAATAAGAAAACCATATTATGCATCAC-CGACACTGGATGGCGGCGTTA |
| Atg1.1 | CTGGGGAAACAGAGAACAGT |

**Supplementary experimental procedures**

**Construction of Δ*atg1*Δ*pex3* strains**

*ATG1* gene was deleted in Δ*pex3* cells by replacing the open reading frame with nourseothricin resistance gene {Goldstein, 1999 #32}. The *atg1::NatMX4* DNA fragment was amplified with ATG1up and ATG1down primers (Table S2) using pAG25 {Goldstein, 1999 #32} as template and transformed into Δ*pex3* cells. Correct insertion by homologous recombination was confirmed by colony PCR using Atg1.1 and Pot1.6 primers (Table S2).

**Chronological aging experiment on peroxisome induction medium for *pex* mutants**

Overnight cultures were grown in MM medium containing 0.5% glucose and required amino acids. Those cultures were then diluted twice at OD_600 nm_ = 0.1 in fresh MM containing 0.3% glucose and grown for 8 hours. After the last pre-cultivation step, cells were diluted in MM containing 0.25% ammonium sulfate, 0.05% yeast extract, 0.1% oleic acid and 0.05% Tween 80 and 0.1% glucose. Cultures were incubated at 30°C, 200 rpm. Survival was assayed by counting colony-forming units (CFUs) after 2 days of incubation at 30°C on YPD agar plates. 24 hours after the last dilution (D1) was considered as 100% of survival. The results shown are mean values and standard error of mean. Statistical analyses were determined using two-way ANOVA. A p value of less than 0.05 was considered as a significant difference.
